# Total cholesterol content of erythrocyte membranes is associated with the severity of coronary artery disease and the therapeutic effect of rosuvastatin

**DOI:** 10.3109/03009734.2012.672345

**Published:** 2012-10-30

**Authors:** Yucheng Zhong, Hongxia Tang, Qiutang Zeng, Xiang Wang, Guiwen Yi, Kai Meng, Yi Mao, Xiaobo Mao

**Affiliations:** ^1^Department of Cardiology, Institute of Cardiovascular Disease, Union Hospital, Tongji Medical College, Huazhong University of Science and Technology, Wuhan, 430022, China; ^2^Department of Pediatric Infectious and Immunological Diseases, Wuhan Children's Hospital, Wuhan, 430016, China

**Keywords:** Coronary artery disease, cholesterol content, erythrocyte membranes, rosuvastatin

## Abstract

**Introduction.:**

Numerous studies suggest that total cholesterol content of erythrocyte membranes (CEM) might play a critical role in atherosclerotic plaque progression and instability. However, the exact role of CEM in atherosclerosis remains obscure. Our study was designed to investigate the association between CEM and the severity of coronary artery disease (CAD), and to assess the effect of rosuvastatin on CEM levels.

**Methods.:**

CEM levels were assessed in 136 participants, including acute coronary syndrome (ACS) (non-ST-segment elevation ACS (NSTEACS) and ST-segment elevation myocardial infarction (STEMI)), stable angina pectoris (SAP), and controls. The Gensini score was used to estimate the severity of CAD. Additionally, 54 patients with CAD were medicated with rosuvastatin, 5 or 10 mg once daily, and then checked at 6 months.

**Results.:**

The highest level of CEM was found in the STEMI group, followed by the NSTEACS, the SAP, and the control groups. Gensini score in group IV (CEM > 141.6 μg/mg) was markedly higher compared with group I (CEM ≤77.6 μg/mg). Gensini scores in group II (77.6 < CEM ≤111.1 μg/mg) and group III (111.1 < CEM ≤141.6 μg/mg) were also higher than in group I (all *P* < 0.001). Furthermore, a positive correlation was found between CEM levels and Gensini score (*r* = 0.714, *P* < 0.001). CEM levels were dose-dependently reduced by rosuvastatin therapy.

**Conclusions.:**

CEM levels are positively associated with the severity of CAD, meaning that CEM might contribute to the development of CAD. Importantly, rosuvastatin could decrease CEM levels in patients with CAD and might effectively help to attenuate the progression of CAD.

## Introduction

It is well known that the accumulation of cholesterol in atherosclerotic plaques can lead to plaque instability and subsequently result in acute coronary syndrome (ACS) (1,2). Previous studies have suggested that cholesterol within plaques is mainly derived from apoptotic/necrotic foam cells (2). The cholesterol in foam cells is mostly esterified (3). However, the proportion of free cholesterol in atherosclerotic plaques is markedly high (4). Therefore, it is logical to suggest that cholesterol present in plaques might be derived from other sources. Arbustini et al. and Kolodgie et al. observed that erythrocyte membranes were present in the necrotic core of advanced atherosclerotic plaques (5,6). Further studies have shown that erythrocyte membranes contribute to a significant increase of cholesterol accumulation in atherosclerotic plaques (7), since these membranes contain large amounts of cholesterol (6). Thus, it is reasonable to postulate that erythrocytes are an important source of cholesterol in plaques.

Recently, accumulating clinical studies have shown that the cholesterol content of erythrocyte membranes (CEM) in ACS patients is significantly increased compared to patients with stable angina pectoris (SAP) and is considered a potential marker of coronary artery disease (CAD) activity (1,8–13). Additionally, CEM contributes to necrotic core expansion and then results in atherosclerotic plaque vulnerability. However, the relationship between the level of CEM and the angiographic degree of coronary atherosclerosis is a controversial issue.

The aim of this study was to investigate the relationship between CEM levels and the severity of coronary artery stenosis in Chinese patients with CAD, as assessed by Gensini score, and to estimate the therapeutic effect of rosuvastatin on CEM levels in patients with CAD.

## Materials and methods

### Participants

A total of 106 consecutive patients who were admitted to the Cardiology Department of Union Hospital Wuhan, Hubei, PR China, from April 2010 to October 2010 for assessment of angina pectoris were recruited for the study. Of these, 72 had ACS (31 ST-segment elevation myocardial infarction (STEMI) and 41 non-ST-segment elevation ACS (NSTEACS); the latter consisted of non-STEMI and unstable angina), and 34 had SAP. Diagnosis of STEMI, non-STEMI, unstable angina, and SAP was performed according to established guidelines (14–17). In addition, 30 patients with atypical chest pain and normal coronary arteries on angiography were considered as controls. All participants underwent coronary angiography after admission. Cardiovascular-interrelated factors, such as age, gender, body mass index (BMI), blood pressure, heart rate (HR), and ejection fraction (EF), etc., were estimated via physical examination, electrocardiogram (ECG), and ultrasonic cardiography (UCG). All participants were divided into four groups according to the quartile of CEM levels; the lowest quartile group was considered as control.

Patients with CAD (*n* = 54), who were not receiving hypolipidemic therapy before admission, were medicated with rosuvastatin, 5 or 10 mg once daily, after initial blood sampling, and then followed for 6 months. The dose of rosuvastatin used was decided by plasma low-density lipoprotein cholesterol (LDL-C) levels (cut point 2.6 mmol/L). After that the patients were allocated into two groups: 5 mg rosuvastatin group (low-dose group, *n* = 25) and 10 mg rosuvastatin group (high-dose group, *n* = 29).

Exclusion criteria included patients with a history of excessive alcohol intake, hematological, liver, renal, or thyroid diseases, infectious or autoimmune diseases, familial hyperlipidemia, cancer, and those having undergone surgical procedures in the preceding 3 months. Patients who were receiving treatment with anti-inflammatory drugs or hormone replacement therapy were not included in the study. Furthermore, patients with abnormal red blood cell (RBC) counts (<4.0 and > 5.5 × 10^12^/L for men and < 3.5 and > 5.0 × 10^12^/L for women) and/or abnormal hemoglobin (Hb) levels (<120 and > 160 g/L for men and < 110 and > 150 g/L for women) were also excluded from the study. The study was approved by the Ethics Committee of Tongji Medical College of Huazhong University of Science and Technology, and all participants gave written informed consent prior to study entry.

### Angiographic analyses

Angiographic images were visually evaluated by two experienced cardiologists, who were not aware of the patients' clinical and biochemical results, to assess the extent and severity of CAD. A coronary artery was defined as ‘diseased’ in the presence of ≥ 50% luminal narrowing (18). The extent of the atherosclerotic disease in the coronary artery tree was assessed by vessel score (1), and the severity of CAD was assessed by Gensini score as previously described (19).

### Laboratory analyses

From all participants, venous blood samples were obtained after a 12h-overnight fast, prior to coronary angiography. Blood specimens for CEM were collected in standard vacutainer tubes containing citrate, then centrifuged at 450 *g* for 10 min at 4°C, and then the plasma and buffy coat were carefully discarded by aspiration. The remaining RBCs were resuspended and washed three times at 450 *g* for 5 min using 0.15 mol/L sodium chloride, and subsequently lysed in 30 volumes of hemolysis buffer (10 mM Tris-HCl, 1 mM EDTA, pH 7.4). The membranes were separated from the hypotonic solution containing hemolyzed RBCs by centrifugation at 27,000 *g* for 15 min at 4°C; washings with the hypotonic buffer were repeated at least three times until a white/pink−pale pellet consisting of hemoglobin-free erythrocyte ‘ghosts’ was collected (1,9,20). Erythrocyte ghosts, which were redissolved in 1 mL of phosphate-buffered saline (PBS) (pH 7.4), were stored at -60°C until further analysis. Membrane protein concentration was measured by the Bicinchoninic acid (BCA) protein assay kit (ThermoFisher, Rockford, USA) (21), and the sensitivity of the assay was 5 mg/L. RBC membrane lipid extraction was performed following the procedure described by Folch et al. (22), and total cholesterol, including free and esterified cholesterol, was determined using a commercial enzymatic assay (DiaSys Diagnostic Systems GmbH, Holzheim, Germany) according to the manufacturer's instruction (1). The lower limit of detection in blood serum was 30 mg/L, and the manufacturer's reported intra- and inter-assay precision, expressed as a percentage of the coefficient of variation (CV, %), was < 2% in both cases. In brief, a six-point calibration curve was obtained by diluting the standard solution provided in the kit, the absorbance of each sample was measured against a blank at 500 nm, and the result was plotted against the calibration curve to obtain the quantity of total cholesterol. All samples were measured in duplicate. Results were expressed as micrograms of total membrane cholesterol per milligram of membrane protein (μg/mg).

All other biochemical measurements, including serum total cholesterol, triglyceride, high-density lipoprotein cholesterol (HDL-C), LDL-C, apolipoprotein A-I (ApoA-I), apolipoprotein B (ApoB), lipoprotein(a), fasting glucose, creatinine, uric acid, and high-sensitivity C-reactive protein (hs-CRP), were carried out by the biochemical laboratory of our cardiovascular institute using standard methods.

### Statistical analyses

Continuous variables are presented as mean ± standard deviation (SD) or as medians and interquartile ranges if the distributions were non-normal, while categorical data are presented as percentages. The Shapiro–Wilk test was used to assess the normality of distribution of continuous variables. For comparisons of two groups, continuous variables were tested using the independent samples *t* test for normally distributed data and the Mann–Whitney *U* test for non-normally distributed data; the chi-square test was used for categorical variables. When three or more groups were compared, one-way ANOVA (normally distributed data) and Kruskal–Wallis tests (skewed variables) were used. The Spearman rank correlation coefficient was used to assess the relationship between CEM levels and other variables. Statistical analysis was carried out using SPSS 13.0 (SPSS Inc., Chicago, IL). A *P* value of < 0.05 was considered statistically significant.

## Results

### Baseline characteristics

Table I summarizes the general characteristics of the study subjects. Patients in the SAP and NSTEACS groups were markedly older than controls. A decreased left ventricular ejection fraction in patients with ACS was shown compared with controls, whereas no difference was found between patients with SAP and controls. A prevalence of smoking was significantly higher in patients with ACS compared to patients with SAP. However, there were no differences in hypertension, diabetes mellitus, dyslipidemia, or family history between patients with ACS and SAP. Compared to patients with ACS, the use of aspirin and statins was more common in patients with SAP before admission. Compared with the control group, a significant decrease of HDL-C levels and an obvious increase of lipoprotein(a) and hs-CRP levels were observed in patients with CAD. Nonetheless, other biochemical results, including total cholesterol, triglyceride, LDL-C, ApoA-I, ApoB, fasting glucose, creatinine, uric acid, and hemoglobin levels were similar between CAD patients and controls.

**Table I.** Demographic and clinical data of the participants at admission.

	SAP (*n* = 34)	NSTEACS (*n* = 41)	STEMI (*n* = 31)	Controls (*n* = 30)	*P* value
Age, years	61.9 ± 7.7	60.3 ± 7.9	54.2 ± 8.6	54.6 ± 8.2	0.002^a^
Men/women, *n*	23/11	35/6	26/5	24/6	0.246
BMI, kg/m^2^	24.1 ± 2.2	24.1 ± 1.6	24.6 ± 2.2	24.0 ± 2.1	0.803
SBP, mmHg	133.7 ± 14.6	127.7 ± 11.2	126.8 ± 14.1	128.1 ± 10.6	0.279
DBP, mmHg	80.9 ± 9.4	77.2 ± 7.4	80.1 ± 9.2	81.5 ± 8.5	0.355
HR, bpm	65.6 ± 7.0	68.9 ± 8.1	73.5 ± 12.5	67.9 ± 6.0	0.206
EF (%)	68.8 ± 6.5	60.7 ± 6.3	54.2 ± 6.6	68.2 ± 4.0	0.001^a^
History of PCI (%)	5	2	3	0	0.725
History of CABG (%)	0	0	0	0	0
Risk factors					
Hypertension, *n* (%)	23 (68%)	27 (66%)	15 (48%)	0	0.211
Diabetes mellitus, *n* (%)	9 (26%)	11 (27%)	10 (32%)	0	0.844
Dyslipidemia, *n* (%)	6 (18%)	8 (20%)	5 (16%)	0	0.932
Smoking, *n* (%)	14 (41%)	25 (61%)	22 (71%)	11 (37%)	0.017^b^
Family history, *n* (%)	7 (21%)	9 (22%)	6 (19%)	0	0.964
Medications					
Aspirin, *n* (%)	21 (62%)	11 (27%)	7 (23%)	0	0.001^b^
Clopidogrel, *n* (%)	3 (9%)	2 (2%)	2 (7%)	0	0.790
Beta-blockers, *n* (%)	15 (44%)	12 (29%)	6 (19%)	0	0.093
ACE inhibitors, *n* (%)	9 (26%)	13 (32%)	8 (26%)	0	0.825
ARB, *n* (%)	7 (21%)	6 (15%)	5 (16%)	0	0.783
CCB, *n* (%)	12 (35%)	10 (24%)	8 (26%)	0	0.542
Statins, *n* (%)	16 (47%)	9 (22%)	6 (19%)	0	0.021^b^
Nitrates, *n* (%)	5 (15%)	4 (10%)	2 (7%)	0	0.545
Angiographic analysis					0.612
Non-significant disease	3 (9%)	3 (7%)	2 (7%)	30 (100%)	
1-vessel disease	12 (35%)	10 (24%)	9 (29%)	0	
2-vessel disease	14 (41%)	15 (37%)	10 (32%)	0	
3-vessel disease	5 (15%)	13 (32%)	10 (32%)	0	
LMS disease	2 (6%)	5 (12%)	2 (7%)	0	0.552
Biochemistry					
Total cholesterol, mmol/L	4.36 ± 0.77	4.29 ± 0.72	4.72 ± 1.02	4.43 ± 0.73	0.335
Triglyceride, mmol/L	1.62 (1.05–2.23)	1.38 (1.07–2.00)	1.63 (1.02–2.25)	1.60 (0.98–2.11)	0.940
HDL cholesterol, mmol/L	1.24 ± 0.24	1.12 ± 0.16	1.08 ± 0.21	1.26 ± 0.27	0.021^a^
LDL cholesterol, mmol/L	2.36 ± 0.63	2.32 ± 0.60	2.64 ± 0.86	2.31 ± 0.59	0.346
ApoA-I, mmol/L	1.06 ± 0.17	1.03 ± 0.14	1.02 ± 0.15	1.13 ± 0.21	0.189
ApoB, mmol/L	0.93 ± 0.15	0.95 ± 0.18	0.96 ± 0.19	0.94 ± 0.18	0.958
Lipoprotein (α), mg/L	38.5 (27.3–57.0)	55.9 (31.3–65.6)	51.9 (35.9–80.4)	34.7 (21.6–52.1)	0.018^c^
Glucose, mmol/L	4.99 (4.25–6.12)	4.90 (4.40–6.10)	5.30 (4.55–6.34)	4.80 (4.42–5.20)	0.375
Creatinine, μmol/L	74.6 ± 8.5	76.4 ± 9.7	77.7 ± 9.5	73.0 ± 9.8	0.595
Uric acid, μmol/L	338.1 ± 81.9	351.1 ± 50.4	367.1 ± 76.8	334.4 ± 61.7	0.494
Hemoglobin, g/L	132.1 ± 11.4	137.8 ± 11.0	136.3 ± 10.9	136.1 ± 11.0	0.213
hs-CRP, mg/L	3.33 (2.92–3.85)	4.58 (3.36–6.71)	5.77 (4.86–9.43)	3.55 (2.59–4.04)	<0.001^c^

Values are expressed as mean ± standard deviation (SD) or median and interquartile range for continuous variables, and as % for categorical variables.

^a^One-way ANOVA.

^b^Chi-square test.

^c^Kruskal–Wallis test.

ACE = angiotensin-converting enzyme; ApoA-I = apolipoprotein A-I; ApoB = apolipoprotein B; ARB = angiotonin II receptor blocker; BMI = body mass index; CABG = coronary artery bypass graft; CCB = calcium antagonists; DBP = diastolic blood pressure; EF = ejection fraction; HDL = high-density lipoprotein; HR = heart rate; hs-CRP = high-sensitivity C-reactive protein; LDL = low-density lipoprotein; LMS = left main stem; NSTEACS = non-ST-segment elevation acute coronary syndrome; PCI = percutaneous coronary intervention; SAP = stable angina pectoris; SBP = systolic blood pressure; STEMI = ST-segment elevation myocardial infarction.

Table II depicts the characteristics of participants classified according to the quartile of CEM levels.

**Table II.** Characteristics of all participants classified according to the quartile of CEM levels.

	Quartile of CEM level (μg/mg), groups I - IV 3.			
	≤77.6 (*n* = 34)	77.6–111.1 (*n* = 34)	111.1–141.6 (*n* = 34)	>141.6 (*n* = 34)
Age, years	57.1 ± 9.3	59.0 ± 8.4	57.4 ± 7.8	58.4 ± 9.2
Men/women, *n*	27/7	26/8	27/7	28/6
BMI, kg/m^2^	24.2 ± 2.5	24.1 ± 1.7	24.0 ± 1.9	24.3 ± 1.8
Hypertension, *n* (%)	17 (50%)	17 (50%)	16 (47%)	21 (62%)
Diabetes mellitus, *n* (%)	5 (15%)	6 (18%)	11 (32%)	9 (26%)
Dyslipidemia, *n* (%)	4 (12%)	6 (18%)	9 (26%)	2 (6%)
Smoking, *n* (%)	14 (41%)	14 (41%)	20 (59%)	22 (65%)
Family history, *n* (%)	3 (9%)	3 (9%)	7 (21%)	9 (26%)
Total cholesterol, mmol/L	4.18 ± 0.72	4.37 ± 0.66	4.60 ± 0.91	4.63 ± 0.94
Triglyceride, mmol/L	1.29 (0.96–2.41)	1.52 (0.94–2.03)	1.68 (1.18–2.17)	1.52 (1.08–2.18)
HDL cholesterol, mmol/L	1.15 ± 0.29	1.14 ± 0.20	1.11 ± 0.18	1.12 ± 0.22
LDL cholesterol, mmol/L	2.07 ± 0.56	2.40 ± 0.55	2.67 ± 0.78^b^	2.50 ± 0.71^a^
ApoA-I, mmol/L	1.07 ± 0.19	1.10 ± 0.14	1.04 ± 0.15	1.03 ± 0.19
ApoB, mmol/L	0.89 ± 0.18	0.95 ± 0.15	1.00 ± 0.17	0.91 ± 0.20
Lipoprotein (α), mg/L	27.2 (20.5–41.4)	43.8 (32.0–58.3)^b^	54.6 (32.7–78.4)^c^	58.1 (42.5–69.3)^c^
Glucose, mmol/L	4.65 (4.40–5.76)	4.85 (4.71–6.06)	4.95 (4.45–6.06)	5.47 (4.40–6.88)
Creatinine, μmol/L	74.4 ± 9.7	76.9 ± 14.5	69.4 ± 10.4	77.3 ± 13.2
Uric acid, μmol/L	336.2 ± 65.9	333.2 ± 62.2	331.8 ± 51.9	334.3 ± 81.9
Hemoglobin, g/L	134.6 ± 10.8	137.7 ± 13.8	134.7 ± 10.4	143.3 ± 19.8
hs-CRP, mg/L	2.87 (2.05–3.17)	3.49 (3.21–3.83)^c^	4.76 (3.82–5.48)^c^	8.21 (5.71–9.60)^c^
Gensini score	3 (0–16.6)	18.5 (6.9–41.9)^c^	42 (24.3–56.0)^c^	70.3 (48.4–99.0)^c^

Values are expressed as mean ± standard deviation (SD) or median and interquartile range for continuous variables, and as % for categorical variables. *P* values were calculated using chi-square test, one-way ANOVA, or Kruskal–Wallis test.

^a^ *P* < 0.05;

^b^ *P* < 0.01;

^c^ *P* < 0.001, compared with the CEM level ≤77.6 μg/mg group.

ApoA-I = apolipoprotein A-I; ApoB = apolipoprotein B; BMI = body mass index; HDL = high-density lipoprotein; hs-CRP = high-sensitivity C-reactive protein; LDL = low-density lipoprotein.

**Figure 1.**
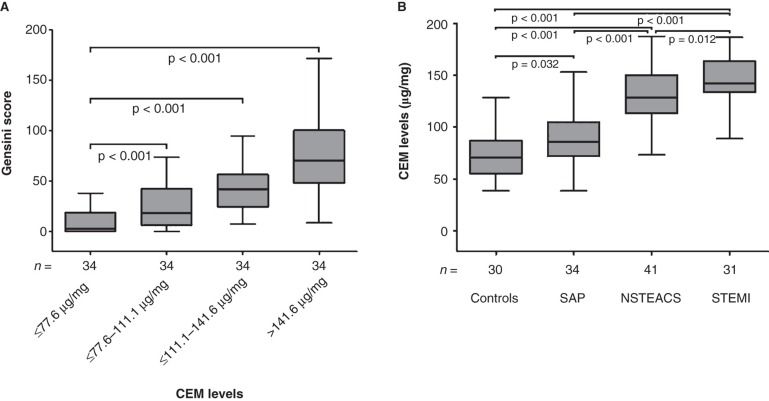
A: Gensini score in patients within different groups according to the quartile of CEM level. Horizontal line in the box plots represents the median value; the boxed area is the interquartile range, and the whiskers indicate minimum and maximum. B: CEM levels in controls and patients with stable angina pectoris (SAP), non-ST-segment elevation acute coronary syndrome (NSTEACS), and ST-segment elevation myocardial infarction (STEMI). CEM = total cholesterol content of erythrocyte membrane.

Gensini score in group IV (median 70.3, interquartile range 48.4–99), group III (42, 24.3–56), and group II (18.5, 6.9–41.9) were markedly higher than in group I (3, 0–16.6) (all *P* < 0.001) (Figure 1A). LDL-C of groups III and IV were higher than that of group I, respectively (all *P* < 0.05). Lipoprotein(a) and hs-CRP levels in groups II, III, and IV were higher compared to group I, respectively (all *P* < 0.01).

### Total cholesterol content of erythrocyte membranes

Significantly higher CEM levels were observed in the STEMI group (142.5, 134.2–163.8 μg/mg, *P* < 0.001), the NSTEACS group (128.5, 115.6–145.5 μg/mg, *P* < 0.001), and the SAP group (86.1, 72.9–103 μg/mg, *P* < 0.05) compared with the control group (70.6, 55.7–86.6 μg/mg). Importantly, CEM levels in patients with STEMI were obviously increased compared to patients with NSTEACS and SAP, and CEM levels in patients with NSTEACS were markedly higher than in patients with SAP (Figure 1B).

### Rosuvastatin reduces cholesterol content of erythrocyte membranes

**Figure 2.**
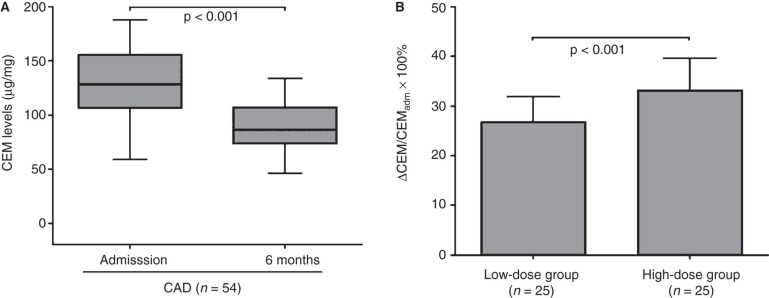
A: CEM levels in patients with coronary artery disease (CAD) on admission and at 6 months. B: The proportion of CEM reduction in patients in the low-dose group and high-dose group. ▵CEM = CEM_adm_ − CEM_6 months_; adm = admission; CEM = total cholesterol content of erythrocyte membrane.

After 6 months of therapy with rosuvastatin, 5–10 mg/day, we found that the CEM levels were significantly decreased compared to admission levels (*P* < 0.001) (Figure 2A). The reduction of CEM in the high-dose group (33.2% ± 4.98%) was markedly expressed in comparison with the low-dose group (26.7% ± 3.90%, *P* < 0.001) (Figure 2B).

### Correlation analysis

We assessed the association of CEM levels with the severity of CAD and several cardiovascular interrelated risk factors. Correlation analysis showed that CEM levels were strongly associated with serum levels of lipoprotein(a) (*r* = 0.312, *P* < 0.001) and hs-CRP (*r* = 0.835, *P* < 0.001). However, there were no significant correlations with serum levels of total cholesterol (*r* = 0.184), triglycerides (*r* = 0.103), HDL-C (*r* = 0.060), LDL-C (*r* = 0.161), ApoA-I (*r* = −0.060), ApoB (*r* = 0.090), fasting glucose (*r* = 0.099), uric acid (*r* = −0.053), nor with age (*r* = −0.128).

**Figure 3.**
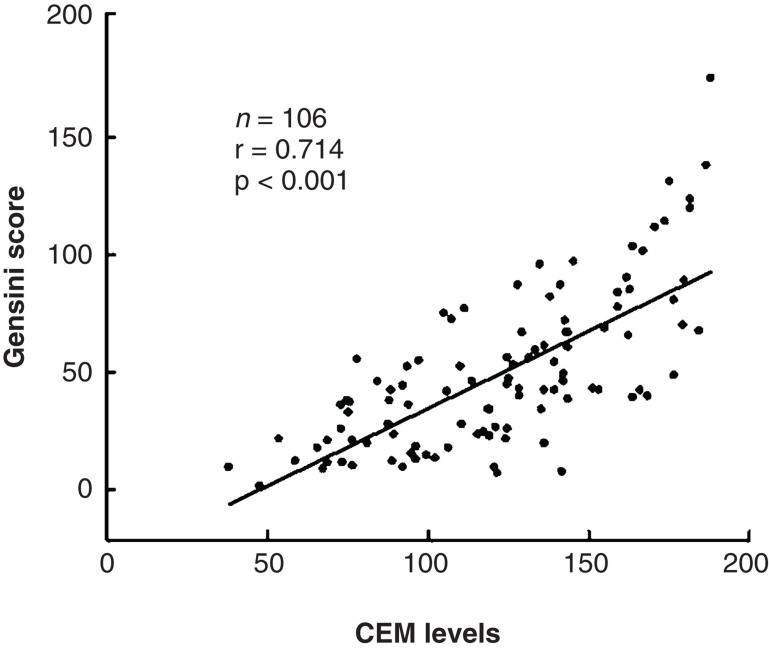
Correlation between CEM levels and coronary angiographic Gensini score in patients with coronary artery disease (CAD). CEM = total cholesterol content of erythrocyte membrane.

Interestingly, we found a significant positive correlation between angiographic Gensini score and CEM levels (*r* = 0.714, *P* < 0.001) (Figure 3). In addition, CEM levels were significantly correlated with vessel score (*r* = 0.471, *P* < 0.001). Our data showed that CEM levels in patients with 2-vessel disease (124.6, 102.6–140.0 μg/mg, *P* = 0.007) and 3-vessel disease (151.6, 136.4–170.7 μg/mg, *P* < 0.001) were significantly higher compared to patients with slight disease (88.9, 77.6–106.7 μg/mg). CEM levels in patients with 1-vessel disease (102.4, 77.3–132.4 μg/mg, *P* = 0.277) were also increased but not statistically significant.

## Discussion

There is increasing evidence that red blood cells, especially their membranes, play an important role in atherosclerotic plaque progression and instability (1,13,23). The stability of the plaque appears to be largely determined by the size of the lipid core (24). Moreover, several studies recently suggested that erythrocytes entering the plaque may induce further expansion of atherosclerotic lipid core and promote plaque peroxidation (6,25). Also, another study showed that injection of erythrocytes in aortic atherosclerotic plaques led to a remarkable increase of lipid content in the plaques (26). A clinical role of CEM in atherosclerosis was first reported by Tziakas et al. (1) who found an elevation of CEM in patients with ACS and discussed its implication in the pathophysiological progress of CAD instability. CEM might induce apoptosis of macrophages, formation of foam cells, and growth of lipid core with subsequent plaque rupture or erosion (27).

We show in this study that respective CEM levels in the STEMI, NSTEACS, and SAP groups were significantly higher compared to the control group. Remarkably, the highest level of CEM was found in the STEMI group, followed by the NSTEACS, and the SAP groups. These results are not in agreement with those of Tziakas et al. (1) and Xu et al. (12), who concluded that CEM could be a marker of ACS but not of atherosclerosis. We now propose that CEM levels are higher in patients with STEMI compared to patients with NSTEACS. These data further reinforce the idea that CEM is a marker of CAD instability.

We also report a strong association between CEM levels and the severity of coronary artery stenosis in patients with CAD, as assessed by Gensini score. This was in line with previous studies showing positive correlations between CEM levels and the number of diseased coronary arteries. The coronary atheroma burden was revealed in patients with angiographically based CAD (9,13). On the contrary, Tziakas et al. (1) observed that CEM levels were not associated with the extent and severity of coronary artery disease. Instead, a significant relationship was noted between CEM levels and angiographically complex coronary lesions (1). The reason for the disparity is unclear. It could relate to differences in methods of assessment of the coronary stenosis and the criterion for diagnosis of the diseased artery. Thus, the intricate relationship between CEM and the extent and severity of CAD deserves large-scale clinical trials.

Our data were consistent with previous studies showing that CEM levels correlated with serum hs-CRP levels (1,11,12), the latter being regarded as a biochemical marker of inflammation. Recently, some studies have revealed that serum CRP levels are biochemical markers of ACS, and a significant correlation between CRP level and the severity of atherosclerosis has been documented (28–30). These results further corroborate the idea that CEM is a predictor of CAD instability similarly to previously known biomarkers such as hs-CRP. Additionally, we found no significant correlation between CEM levels and circulating lipids, such as serum total cholesterol, triglycerides, HDL-C, LDL-C, ApoA-I, and ApoB, in patients with CAD. Unexpectedly, a significant, positive relationship was noted between CEM and lipoprotein(a) in our study. Several lines of evidence indicate that lipoprotein(a) is an independent risk factor for patients with CAD, leading to coronary artery disease (31,32). Therefore, these findings suggest that the increase in lipoprotein(a) levels might contribute to augmented CEM levels in CAD, and CEM could accordingly participate in the progress of CAD.

We also demonstrate that CEM levels were significantly decreased in patients with CAD after 6 months of therapy with rosuvastatin, which was dose-dependent. These results are in accordance with previous studies demonstrating that statin treatment reduces CEM levels in patients with CAD and hypercholesterolemia (1,12,20). Although the mechanism of the unloading effect of statins on CEM levels remains obscure, a novel role of statins should be noticed and further validated in a large-scale study.

In conclusion, the present study demonstrates that CEM levels correlated with the severity of coronary artery disease. We have also found a significant relationship between CEM levels and serum hs-CRP and lipoprotein(a) levels. Rosuvastatin treatment reduced CEM levels in patients with CAD dose-dependently. Altogether, CEM may play a pivotal pathogenic role quantitatively and qualitatively in patients with CAD. The reducing effect of statins on CEM levels is a promising new approach in treatment of patients with CAD. However, this study enrolled only a small group of Chinese patients, and a large-scale clinical investigation is warranted. The precise mechanisms underlying our findings and their clinical relevance remain to be clarified.
